# Clinical Approach and Anatomical Results in the Treatment of Retinopathy of Prematurity

**DOI:** 10.5152/eurasianjmed.2024.23140

**Published:** 2024-02-01

**Authors:** Mustafa Yildirim

**Affiliations:** Department of Ophthalmology, Atatürk University School of Medicine, Erzurum, Turkey

**Keywords:** Retinopathy of premature, Laser photocoagulation, Intravitreal Bevacizumab

## Abstract

**Background:**

Retinopathy of prematurity (ROP) is a disease associated with abnormal development of retinal vessels in low birth weight and preterm infants. In this study, it was aimed to show that the disease can be controlled almost perfectly with early diagnosis and treatment in retinopathy developing in premature babies.

**Methods:**

In the study, 66 eyes of 33 patients who needed ROP treatment were evaluated. Babies who met the screening criteria were examined for the first time 3-4 weeks after birth. In the treatment of patients who underwent laser photocoagulation and intravitreal injection, the stage of the disease plus the regression of the disease in the laser group and completion of retinal vascularization in the intravitreal injection group were determined as success criteria.

**Results:**

Laser photocoagulation (LFC) was applied to 54 eyes, intravitreal bevacizumab (IVB) was applied to 8 eyes, and bilateral LFC + IVB was applied to 2 patients in the same session. Since there was no complete regression in the stage of the 2 patients who underwent LFC, an IVB injection was made into one eye of a patient and both eyes of the other patient. After the treatments, the disease regressed and retinal macular traction did not occur.

**Conclusion:**

It has been indicated that timely intervention in patients with treatment indications after regular screening for ROP can prevent possible blindness.

**Main Points**

- Retinopathy of prematurity (ROP) is a serious disease that can cause permanent vision loss in infants.
- The baby should be followed from the fourth week after birth, and the treatment should be provided without delay according to the course of the disease.
- Successful results can be achieved with laser and intravitreal bevacizumab treatments depending on the type of ROP.

## Introduction

Retinopathy of prematurity (ROP) is a disease associated with abnormal proliferation of retinal vessels in low birth weight and preterm infants. It was first reported by Terry in 1942 and it causes severe vision loss if left untreated.^1,2^

Retinopathy of prematurity is one of the most important causes of preventable blindness and vision loss in infants. With the early diagnosis and treatment of patients, possible blindness can be prevented. Especially low birth weight and gestational age are known as the most important risk factors for the disease.^3,4^

In a multicenter study conducted in Turkey, it was reported that premature babies with a gestational age of ≤34 and a birth weight below 1700 g should be screened for ROP.^5^

Laser photocoagulation (LFC) therapy has been the treatment of choice for type 1 ROP for many years.^6^ Thus, vascular endothelial growth factor (VEGF) in the retina-producing cells is destroyed. VEGF plays a significant role in disease progression in ROP.^7^

Anti-VEGF drugs are used in the emergency treatment of aggressive ROP. Intravitreal bevacizumab (IVB) was certified for use in metastatic colon cancer by the U.S. Food and Drug Administration in 2004. Intravitreal bevacizumab, off-label, in the treatment of ROP, and alone, with LFC or vitrectomy, is used.^8,9^

In this study, it is tried to present the results of laser treatment and IVB injection in premature babies who need treatment in ROP and to convey the experience of a physician who has just started ROP treatment.

## Material and Methods

A total of 33 babies born before 35 weeks of gestation who needed treatment during ROP follow-up in the ophthalmology outpatient clinic and the Pediatric Health and Diseases Neonatal Intensive Care Service between December 2019 and November 2022 were included in the study. Patients with other eye pathologies (cataract, corneal, and retinal diseases) other than ROP were excluded from the study. Ethics committee approval was received from the Ethics Committee of Atatürk University Medical Faculty (B.30.2.ATA.0. 01.00/141, Date: 27.01.2022). According to the criteria set by the American Academy of Pediatrics, the American Pediatric Ophthalmology and Strabismus Association, and the American Academy of Ophthalmology, the first fundus examinations were performed in the third week after birth in babies born before 33 weeks or weighing less than 1500 g. The first fundus examinations of babies older than 33 weeks but whose clinical course did not go unstable were also performed at 3-4 weeks post partum.^10^

Forty-five minutes before the examination, 1% tropicamide and 2.5% phenylephrine drops were dropped into both eyes 3 times at 5-minute intervals, and the pupils were dilated. A sterile lid speculum, which is used separately for each patient, was inserted, and the anterior segment structures were evaluated first. Then the retinal posterior pole and scleral buckling were performed using a 20 diopter double aspheric lens, and the retinal periphery was evaluated. All examinations were performed by the same physician (MY). Zones and stages of the cases were recorded according to the criteria determined by the International ROP Committee (International Classification of Retinopathy of Prematurity).^11^ Observed examination findings were noted in the ROP follow-up book and, if hospitalized in the neonatal intensive care unit, in the patient’s file.

Cases with type 1 ROP and APROP (Aggressive Posterior ROP) were treated according to the ETROP study.^12^ In the study, the patients were evaluated in 2 groups. group 1: was classified as “plus” disease with stage 2 or 3 in zone 2, and group 2: as APROP. The ablation procedure (200-300 mW; 0.2-0.4 seconds) was applied to the entire avascular retina at half-spot intervals with conventional LFC in the babies in group 1. In group 2, IVB was administered at a dose of 0.625 mg in 0.025 ml, 1.5 mm behind the limbus from the inferior temporal quadrant. Treatments were done under general anesthesia or sedation. Patients who received intravitreal injections were given topical antibiotics for 1 week after the procedure, while patients who received LFC were given topical steroids for 1 week. Birth weight, birth week, treatment weeks, and treatments of the cases were recorded. The families of the babies who needed treatment according to the ETROP criteria were given detailed information about the disease. A handwritten informed consent form was obtained from the parents of the infants, stating that they accept that the disease could lead to blindness despite treatment. After treatment, regression or progression in stage and plus disease was controlled with weekly follow-ups for the first month. Regression in plus disease and staging and absence of retinal traction or retinal detachment were determined as success criteria in treatment. Follow-up and treatment procedures were performed by the same physician (MY).

## Results

The patient files of 726 premature babies born before the 35th week of gestation between December 2019 and November 2022 were evaluated retrospectively. A total of 66 eyes of 33 babies [18 boys (54.5%) and 15 girls (45.5%)] who needed treatment were included in the study. The birth weight of the patients was between 640 and 1170 g, and the mean was 841.4 ± 12.19 g. The birth weeks of the babies were between 22 and 31 weeks, and it was determined as 25.94 ± 0.16 weeks. Gender, week of birth, birth weight, and distribution of babies with indications for treatment in ROP are shown. (Table 1)

Laser photocoagulation was applied to 54 eyes, IVB to 8 eyes, and bilateral LFC + IVB to 2 patients in the same session. Since there was no complete regression in stage in 2 patients who underwent LFC, IVB injection was performed in 1 eye of a patient and both eyes of the other patient. Initial treatment approaches for the eyes are shown in Table 2. 

All of the first group patients underwent LFC, but in 2 patients, the stage did not regress sufficiently, so additional IVB therapy was applied to the patients (3 eyes in total). In 1 patient from group 1, additional LFC was applied to the patient because there was not enough regression in the stage and plus in both eyes. Two patients in group 1 had vitreous turbidity, and the retinal periphery could not be evaluated clearly, so LFC + IVB injection was performed in both eyes in the same session. Eyes requiring additional treatment are shown in Table 3. 

Treatment implementation weeks for groups of patients treated with premature retinopathy are shown in Table 4.

The patients were followed up by the same physician (MY) for an average of 13 months (3-30). While additional IVB was applied to 2 patients in group 1 and additional LFC was performed on 1 patient, no additional treatment was applied to 4 patients in group 2. The patients in group 1 were followed until the stage and plus disease regressed, and the patients in group 2 were followed until retinal vascularization was completed. One patient in group 2 died due to advanced respiratory failure during the follow-up in the intensive care unit. No retinal detachment or macular traction was observed in the follow-up of the patients whose treatment was completed in both groups. After the treatments applied, anatomical success was achieved in 64 eyes (100%).

## Discussion

Retinopathy of prematurity is one of the most widespread causes of blindness and vision loss, despite treatment in childhood. In parallel with the development of intensive care units in recent years and the increase in the number of babies that can be kept alive with appropriate treatments, the incidence of ROP is increasing.^13^

In the treatment of ROP, treatment methods and treatment indications for prethreshold disease were researched. In the multicenter ETROP study, prethreshold disease was divided into 2 groups as high-risk and low-risk: type 1 ROP and type 2 ROP, respectively. The presence of “plus” disease with any stage in zone 1, stage 3 without “plus” disease in zone 1, and “plus” disease with stage 2 or 3 in zone 2 were defined as type 1 ROP disease. Patients with type 1 ROP and threshold disease were treated with LFC, and the results were compared. It has been observed that patients with type 1 ROP have less negative retinal results and better visual acuity than those with threshold disease, and it has been reported that patients with type 1 ROP need to be treated.^12^ As a result of the studies, the ablation procedure of the avascular retinal area with trans-pupillary LFC is first applied in the treatment of ROP to the extent of the indication.

Gunay et al^14^ have achieved anatomical achievement in 84.7% of the cases at the end of 1 year in their study evaluating the effectiveness of LFC treatment in type 1 ROP, threshold disease, and APROP.

Arvas et al. have reported that they applied diode LFC treatment to 452 eyes with zone 1 type 1 ROP, that they protected the macula at a rate of 98%, and that LFC was an effective and safe treatment in zone 1 ROP.^15^ The success rate of LFC treatment is lower in cases in zone 1 than in cases in zone 2, as said in the literature. Moreover, it has been stated that patients with Zone 1 Retinopathy of Prematurity (ROP) develop significant visual field loss with LFC due to the avascular field width.^16^ If the ROP does not regress in spite of multiple LFC treatments, surgical treatment may be required.^17,18^ Consequently, new treatment alternatives are being researched in the treatment of ROP.

Intravitreal bevacizumab is currently used off-label in the treatment of ROP alone, in combination with LFC or vitrectomy.^8,9^ In the acute phase of the disease, it slows the progression of the disease, giving time for retinal vascularization to continue. Owing to the large molecular weight of IVB, its transmission to the systemic circulation is less than other anti-VEGF drugs, as indicated in animal studies.^19^ Half-life is relatively prolonged when LFC and IVB are administered together.^20,21^ The most significant disadvantage of IVB is the lack of definitive data on the safety of its use in newborns. Even though retinal vessel progression continues after IVB, the fact that extreme peripheral retina cannot be fully vascularized is another disadvantage.^22^ This situation poses a risk for the development of ischemia in the peripheral retina. In addition, the long-term results of IVB are not yet known. It is reported that bilateral tractional retinal detachment developed in a patient treated with IVB 3 years later by Hajrasouliha et al.^23^

When the recurrence rates of the patients who underwent LFC and IVB were examined in the study, no recurrence was observed in the IVB group, while additional IVB treatment was applied in 2 patients in the LFC group, since the disease did not regress. In 1 patient in group 1, the disease was brought under control by applying additional laser treatment to the areas (without laser scar) in the avascular retinal area. Due to the rapid course of the disease in group 2 and the large avascular retinal area, IVB was considered as the first treatment choice in this group of patients.

In the study, it was determined that the progression and severity of the disease increased due to the low birth week of the patient. The width of the avascular area may cause the disease to progress more rapidly with the increase of VEGF secretion secondary to ischemia.

In our country, LFC, IVB, and their combined applications are currently used in the treatment of ROP. In recent years, IVB treatment has started to be preferred more, especially in terms of ease of application, duration of the procedure, and rapid response to treatment. However, in this study, the main treatment approach was determined as LFC because of the long-term problems that IVB may cause in infants and the possibility of not completing the vascularization of the retinal periphery. Intravitreal bevacizumab treatment was preferred, especially in patients with APROP. As the infants grow, the examination conditions become more difficult, and the inability to predict how long the avascular retinal area will be followed up constitutes the difficulty of using IVB except for APROP patients.

Laser photocoagulation treatment in ROP is unfortunately a treatment approach that ophthalmologists have difficulty in applying or do not prefer, considering the difficulty of the application technique and the length of the procedure. However, anatomical success is achieved in the majority of babies who undergo laser treatment in ROP, and patient follow-up can be done more easily. Although LFC treatment left a defect in the peripheral visual field, it was the first choice in the treatment of type 1 ROP because it provided the preservation of central vision in the patient and was safer in the follow-up of the disease.

In some of the patients who received IVB as their first treatment choice, LFC treatment may be required when the disease does not show regression. The financial and moral difficulties of patient referrals, the search for a center to treat the patient during the referral process, and the loss of time due to this constitute a process that will cause serious problems for both the patient and the physician. LFC treatment, which is on the list of special procedures of the Ministry of Health of the Republic of Turkey, should be encouraged to be carried out by more people in more centers with new regulations that provide more administrative and judicial assurance to the physician.

In this study, the treatment effectiveness of the techniques applied to infants in need of treatment in ROP, regression of the disease and which treatments were applied in the absence of regression were explained. It was aimed to convey the experience of a physician who has just started ROP treatment and to show that the result of ROP in blindness can be prevented to a large extent with careful and timely treatments. It is hoped that this disease, which is undesirable and feared for ophthalmologists, will be shown to have satisfactory results with close follow-up and treatment, and it will be instrumental in helping more physicians to work on this path.

In conclusion, the progression of ROP can be prevented with well-timed and appropriate treatment options. LFC treatment gave effective and promising results in patients with type 1 ROP, which constituted the majority of the patient group in the study. Complete lasering of avascular retinal areas reduced the risk of remaining ischemic retinal areas and prevented the progression of the disease. However, IVB alone or in combination with LFC appears to be an effective treatment option for posteriorly located ROP and APROP. However, the small number of patients who underwent IVB was not sufficient to compare its effectiveness with LFC. Because of the possibility of ischemia in the retina periphery in the long-term follow-up of IVB treatment, LFC treatment was given priority.

## Figures and Tables

**Table 1.** Treated for Premature Retinopathy Patients’ Gender, Birth Week, Weight, and Distribution of Groups

Gender, n (%)		Birth Week [Mean ± SD (Minimum–Maximum)]	Birth Weight [Mean ± SD (Minimum–Maximum)]	Groups, n (%)	
Girl	Boy			Group 1*	Group 2*
15 (45.5%)	18 (54.5%)	25.94 ± 0.16 (22-29)	841.4 ± 12.19 g (640-1170)	58 (87.8%)	8 (12.2%)

SD, standard deviation.

*Group 1 is stage 2 or 3 retinopathy of prematurity with “plus” disease in zone 2, and group 2 is aggressive posterior retinopathy of prematurity.

**Table 2.** Initial Treatment Approaches for the Eyes

	LFC	IVB	LFC + IVB
Group 1	54 (93.1%)	0	4 (6.9%)
Group 2	0	8 (100%)	0

LFC, laser photocoagulation; IVB, intravitreal bevacizumab.

**Table 3.** Patients Requiring Additional Treatment

	LFC	IVB	LFC + IVB
Group 1	2	3	0
Group 2	0	0	0

LFC, laser photocoagulation; IVB, intravitreal bevacizumab.

**Table 4.** Application Weeks of Patients Treated for Retinopathy of Prematurity

	First Week of Treatment	Second Week of Treatment
Group 1	35.6 ± 3.1	39 ± 1.4
Group 2	33.4 ± 2.6	3

## References

[1] Terry TL . Extreme prematurity and fibroblastic overgrowth of persistant vascular sheath behind each crystalline lens. Am J Ophthalmol. 1942;25:203 204. (10.1016/j.ajo.2018.05.024) 30055814

[2] Mc Namara JA Tasman W . Retinopathy of prematurity. Ophthalmol Clin North Am. 1990;3:413 427. (10.5409/wjcp.v5.i1.35)

[3] Onofrey CB Feuer WJ Flynn JT . The outcome of retinopathy of prematurity: screening for retinopathy of prematurity using an outcome predictive program. Ophthalmology. 2001;108(1):27 34; discussion 34. (10.1016/s0161-6420(00)00436-x) 11150259

[4] Seiberth V Linderkamp O . Risk factors in retinopathy of prematurity: a multivariate statistical analysis. Ophthalmologica. 2000;214(2):131 135. (10.1159/000027482) 10720918

[5] Bas AY Demirel N Koc E , et al. 2018 Bas AY Demirel N Koc E , et al. Incidence, risk factors and severity of retinopathy of prematurity in Turkey (TR-ROP study): a prospective, multicentre study in 69 neonatal intensive care units. Br J Ophthalmol. 2018;102(12):1711 1716. (10.1136/bjophthalmol-2017-311789) 29519879 PMC6287567

[6] Connolly BP McNamara JA Sharma S Regillo CD Tasman W . A comparison of laser photocoagulation with trans-scleral cryotherapy in the treatment of threshold retinopathy of prematurity,. Ophthalmology. 1998;105(9):1628 1631. (10.1016/S0161-6420(98)99029-7) 9754168

[7] Smith LE . Through the eyes of a child: understanding retinopathy through ROP the Friedenwald lecture. Invest Ophthalmol Vis Sci. 2008;49(12):5177 5182. (10.1167/iovs.08-2584) 18708611

[8] Chung EJ Kim JH Ahn HS Koh HJ . Combination of laser photocoagulation and intravitreal bevacizumab (Avastin) for aggressive zone I retinopathy of prematurity. Graefes Arch Clin Exp Ophthalmol. 2007;245(11):1727 1730. (10.1007/s00417-007-0661-y) 17690897

[9] Dorta P Kychenthal A . Treatment of type 1 retinopathy of prematurity with intravitreal bevacizumab (Avastin). Retina. 2010;30(4)(suppl):S24 S31. (10.1097/IAE.0b013e3181ca1457) 20224475

[10] Section on Ophthalmology American Academy of Pediatrics, American Academy of Ophthalmology, American Association for Pediatric Ophthalmology and Strabismus. Screening examination of premature infants for retinopathy of prematurity. Pediatrics. 2006;117(2):572 576. (10.1542/peds.2005-2749) 16452383

[11] International Committee for the Classification of Retinopathy of Prematurity. The International Classification of Retinopathy of Prematurity revisited. Arch Ophthalmol. 2005;123(7):991 999. (10.1001/archopht.123.7.991) 16009843

[12] Early Treatment For Retinopathy Of Prematurity Cooperative Group. Revised indications for the treatment of retinopathy of prematurity: results of the early treatment for retinopathy of prematurity randomized trial. Arch Ophthalmol. 2003;121(12):1684 1694. (10.1001/archopht.121.12.1684) 14662586

[13] Mutlu FM Sarici SU . Treatment of retinopathy of prematurity: a review of conventional and promising new therapeutic options. Int J Ophthalmol. 2013;6(2):228 236. (10.3980/j.issn.2222-3959.2013.02.23) 23641347 PMC3633766

[14] Gunay M , Celik G , Ovali F , Yetik H , Aktas A , Gunay BO . One-year clinical outcome after laser treatment for retinopathy of prematurity at a tertiary center in Turkey. Int Ophthalmol. 2015;35(1):27 35. (10.1007/s10792-014-0014-x) 25381161

[15] Arvas S Akar S Sarıcı A Uçar D , et al. Outcomes of diode laser photocoagulation for zone 1 retinopathy of prematurity. Turk Arch Ped. 2012;47:250 253. (10.4274/tpa.137)

[16] Mintz-Hittner HA . Avastin as monotherapy for retinopathy of prematurity. J AAPOS. 2010;14(1):2 3. (10.1016/j.jaapos.2009.12.002) 20227612

[17] Quinn GE Dobson V Barr CC , et al. Visual acuity of eyes after vitrectomy for retinopathy of prematurity: follow-up at 5 1/2 years. The Cryotherapy for Retinopathy of Prematurity Cooperative Group. Ophthalmology. 1996;103(4):595 600. (10.1016/s0161-6420(96)30647-7) 8618758

[18] Repka MX Tung B Good WV , et al. Outcome of eyes developing retinal detachment during the Early Treatment for Retinopathy of Prematurity Study (ETROP). Arch Ophthalmol. 2006;124(1):24 30. (10.1001/archopht.124.1.24) 16401781

[19] Bakri SJ Snyder MR Reid JM Pulido JS Singh RJ . Pharmacokinetics of intravitreal bevacizumab (Avastin). Ophthalmology. 2007;114(5):855 859. (10.1016/j.ophtha.2007.01.017) 17467524

[20] Krohne TU Eter N Holz FG Meyer CH . Intraocular pharmacokinetics of bevacizumab after a single intravitreal injection in humans. Am J Ophthalmol. 2008;146(4):508 512. (10.1016/j.ajo.2008.05.036) 18635152

[21] Kong L Mintz-Hittner HA Penland RL Kretzer FL Chévez-Barrios P . Intravitreous bevacizumab as anti-vascular endothelial growth factor therapy for retinopathy of prematurity: a morphologic study. Arch Ophthalmol. 2008;126(8):1161 1163. (10.1001/archophthalmol.2008.1) 18695118

[22] Mintz-Hittner HA Kretzer FL . Postnatal retinal vascularization in former preterm infants with retinopathy of prematurity. Ophthalmology. 1994;101(3):548 558. (10.1016/s0161-6420(94)31301-7) 8127576

[23] Hajrasouliha AR Garcia-Gonzales JM Shapiro MJ Yoon H Blair MP . Reactivation of retinopathy of prematurity three years after treatment with bevacizumab. Ophthalmic Surg Lasers Imaging Retina. 2017;48(3):255 259. (10.3928/23258160-20170301-10) 28297039

